# Neuronal RING finger protein 11 (RNF11) regulates canonical NF-κB signaling

**DOI:** 10.1186/1742-2094-9-67

**Published:** 2012-04-16

**Authors:** Elaine L Pranski, Nirjari V Dalal, Jeremy H Herskowitz, Adam L Orr, Leah A Roesch, Jason J Fritz, Craig Heilman, James J Lah, Allan I Levey, Ranjita S Betarbet

**Affiliations:** 1 Center for Neurodegenerative Disease, Department of Neurology, Emory University School of Medicine, 615 Michael St., Suite 500, Atlanta, GA, 30322, USA

**Keywords:** A20, E3 ligase, NF-κB, Neuroinflammation, Neuron

## Abstract

**Background:**

The RING domain-containing protein RING finger protein 11 (RNF11) is a member of the A20 ubiquitin-editing protein complex and modulates peripheral NF-κB signaling. RNF11 is robustly expressed in neurons and colocalizes with a population of α-synuclein-positive Lewy bodies and neurites in Parkinson disease patients. The NF-κB pathway has an important role in the vertebrate nervous system, where the absence of NF-κB activity during development can result in learning and memory deficits, whereas chronic NF-κB activation is associated with persistent neuroinflammation. We examined the functional role of RNF11 with respect to canonical NF-κB signaling in neurons to gain understanding of the tight association of inflammatory pathways, including NF-κB, with the pathogenesis of neurodegenerative diseases.

**Methods and results:**

Luciferase assays were employed to assess NF-κB activity under targeted short hairpin RNA (shRNA) knockdown of RNF11 in human neuroblastoma cells and murine primary neurons, which suggested that RNF11 acts as a negative regulator of canonical neuronal NF-κB signaling. These results were further supported by analyses of p65 translocation to the nucleus following depletion of RNF11. Coimmunoprecipitation experiments indicated that RNF11 associates with members of the A20 ubiquitin-editing protein complex in neurons. Site-directed mutagenesis of the myristoylation domain, which is necessary for endosomal targeting of RNF11, altered the impact of RNF11 on NF-κB signaling and abrogated RNF11’s association with the A20 ubiquitin-editing protein complex. A partial effect on canonical NF-κB signaling and an association with the A20 ubiquitin-editing protein complex was observed with mutagenesis of the PPxY motif, a proline-rich region involved in Nedd4-like protein interactions. Last, shRNA-mediated reduction of RNF11 in neurons and neuronal cell lines elevated levels of monocyte chemoattractant protein 1 and TNF-α mRNA and proteins, suggesting that NF-κB signaling and associated inflammatory responses are aberrantly regulated in the absence of RNF11.

**Conclusions:**

Our findings support the hypothesis that, in the nervous system, RNF11 negatively regulates canonical NF-κB signaling. Reduced or functionally compromised RNF11 could influence NF-κB-associated neuronal functions, including exaggerated inflammatory responses that may have implications for neurodegenerative disease pathogenesis and progression.

## Background

The NF-κB transcription factor has important roles in the regulation of programmed cell death, cell proliferation and differentiation, innate and adaptive immune responses, and inflammation [1]. Defects in NF-κB signaling, such as persistent activation, can contribute to the pathology of several cancers and inflammatory diseases [1]. NF-κB also has notable ramifications in the vertebrate nervous system, where the absence of NF-κB activity during development can result in abnormal neurite branching and loss of learning and memory. Notably, persistent activation of NF-κB signaling is associated with chronic neuroinflammation and is implicated in the progression of neurodegenerative diseases [2-11].

To ensure regulated transient activity, NF-κB signaling is tightly controlled with several layers of modulation. Several NF-κB target genes can function as NF-κB inhibitors, such as A20 (also known as TNF-α-induced protein 3, or TNFAIP3), inhibitors of NF-κB (IκBs) and cylindromatosis [12,13]. The ubiquitin-editing protein A20 is a key negative regulator of NF-κB signaling and functions downstream of innate immune receptors such as TNF receptor (TNFR) and Toll-like receptors (TLRs) [14]. A20-deficient mice have widespread inflammation in all organ systems [15], and recent studies have indicated that the regulatory function of A20 is dependent upon interactions with the E3 ligase Itch (also known as AIP4), Tax1 (human T-cell leukemia virus type I) binding protein 1 (TAX1BP1) and RNF11 (RING finger protein 11) [16-19].

RNF11 is a 154-amino acid protein with differential expression in cancer, including breast, pancreatic and colon, as well as in Parkinson disease (PD) [20-23]. RNF11 contains a RING H2 finger domain in its C-terminus, which is a hallmark characteristic of an E3 ubiquitin ligase [24]. RNF11 enhances TGF-β signaling through interactions with Smad4 and Smurf2 [23,25-27]. It is also an essential component of the A20 ubiquitin-editing complex in the periphery and can negatively regulate NF-κB signaling in human monocytic cell lines [19].

**Figure 1:**
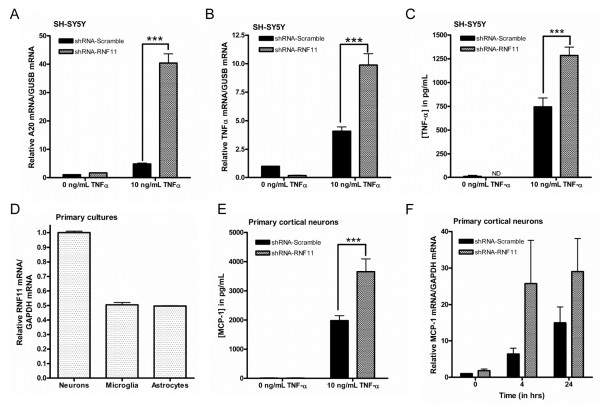
**Knockdown of RNF11 exaggerates inflammatory responses following TNF-α treatment. (A)** and **(B)** Stably transduced SH-SY5Y short hairpin RNA (shRNA)-RNF11 and shRNA-Scramble cells were exposed to 0 or 10 ng/ml TNF-α for 24 hours. The relative amount of mRNA expression in stimulated and unstimulated cells was calculated using quantitative RT-PCR (qRT-PCR) and glucuronidase β (GUSB) as an internal control. **(C)** Stably transduced SH-SY5Y shRNA-RNF11 and shRNA-Scramble cells were exposed to 0 or 10 ng/ml TNF-α for 4 hours before cells were rinsed and fresh media were replaced in the plate. Media were collected after 20 hours and analyzed for TNF-α protein levels using a human TNF-α ELISA. ND, not detectable. **(D)** Murine primary cultures were harvested, and qRT-PCR was used to measure relative RNF11 mRNA levels. **(E)** Murine primary cortical neurons were treated with cytosine arabinoside and transduced with shRNA-Scramble or shRNA-RNF11 lentivirus. Cells were exposed to 0 or 10 ng/ml TNF-α for 4 or 24 hours, and mRNA levels were examined by qRT-PCR. **(F)** Murine primary cortical neurons were transduced with shRNA-Scramble or shRNA-RNF11 lentivirus. Cells were exposed to 0 or 10 ng/ml TNF-α for 4 or 24 hours, and protein levels of monocyte chemoattractant protein 1 (MCP-1) were measured using a mouse MCP-1 ELISA. qRT-PCR values are expressed as mean comparative cycle threshold ± SEM with GUSB (for SH-SY5Y cells) or glyceraldehyde-3-phosphate dehydrogenase (GAPDH) (for primary cultures) levels as an internal control for three independent experiments. ELISA values are expressed as averages of protein levels extrapolated from standard curve analysis from three independent experiments. ^***^P < 0.001.

We have previously demonstrated that RNF11 is differentially expressed throughout the brain and that neurons express higher levels of RNF11 compared to glial cells in tissue [20] (see Figure 1D); however, the function of RNF11 in the nervous system remains to be determined. On the basis of the characterization of RNF11 in monocytic cell lines [19], we hypothesized that neuronal RNF11 modulates NF-κB activity by interacting with the A20 ubiquitin-editing complex. To establish the effects of neuronal RNF11 on NF-κB signaling, targeted knockdown of endogenous RNF11 was employed in human neuroblastoma cells and primary cortical neurons. Reduced RNF11 expression resulted in persistent NF-κB signaling, and association of RNF11 with the A20 ubiquitin-editing protein complex was demonstrated in neuroblastoma cells and primary cortical neuron cultures. Site-directed mutagenesis of RNF11 functional motifs revealed that the myristoylation domain was necessary for RNF11’s association with the A20 ubiquitin-editing protein complex as well as RNF11’s regulation of canonical NF-κB signaling. Furthermore, depletion of RNF11 resulted in aberrant regulation of inflammatory signaling in neuroblastoma cells and primary cortical neurons. Taken together, these studies support a role for RNF11 in regulated activation of canonical NF-κB signaling in neurons and neuroinflammation.

## Methods

### Cell culture

SH-SY5Y and neuroblastoma N2A cells were purchased from the American Type Culture Collection (Manassas, VA, USA). SH-SY5Y cells were cultured in Eagle’s minimal essential medium supplemented with Earle’s balanced salt solution, L-glutamine, 10% fetal bovine serum (FBS) (HyClone Laboratories, Logan, UT, USA), 1% nonessential amino acids (BioWhittaker, Inc, Walkersville, MD, USA) and 1% penicillin/streptomycin (BioWhittaker). N2A cells were cultured in DMEM/Ham’s F-12 nutrient mixture (supplemented with 10% FCS (HyClone Laboratories), 1% nonessential amino acids and 1% penicillin/streptomycin. Primary cortical neurons were prepared from wild-type C57BL/6 mice at embryonic day 18 as previously described [28]. Cells were dissociated by trituration through a Pasteur pipette and plated on 0.25 mg/ml poly-L-lysine-coated dishes in neurobasal medium (Invitrogen, Carlsbad, CA, USA) containing B-27 supplement (Invitrogen), 2 mM L-glutamine and 1% penicillin/streptomycin. Cytosine arabinoside was added at a final concentration of 5 μM on day 3 *in vitro* to control proliferation of nonneuronal cells. After 8 days *in vitro*, neuron-enriched cultures were used for experiments. Other research groups have confirmed that more than 95% of the cells in these culture preparations are positive for neuronal markers [29,30]. Primary microglia and astrocytes were obtained from 1-day-old wild-type C57BL/6 mice as described elsewhere [31,32]. Briefly, cells were dissociated into a suspension by trituration with a Pasteur pipette and plated onto six-well plates coated with 0.05 mg/ml poly-d-lysine and grown in DMEM (Invitrogen) supplemented with 25 mM glucose, 10% heat-inactivated horse serum, 10% heat-inactivated FBS, 2 μM glutamine and 1% penicillin/streptomycin. At the end of day 12 *in vitro*, floating microglia were separated from the stationary cultures and centrifuged at 80 *g* for 5 minutes to obtain a pellet of nearly pure microglia, which were then plated directly into poly-D-lysine-coated dishes. All cultures were maintained at 37°C in 5% CO_2_.

### Antibodies

The following antibodies were used: A20 (ab13597; Abcam, Cambridge, MA, USA), β-actin (ab6276; Abcam), Flag (F1804; Sigma-Aldrich, St Louis, MO, USA), histone 1 (MAB052; Millipore, Billerica, MA, USA), Itch (611198; BD Transduction Laboratories, San Diego, CA, USA), p65 (for immunocytochemistry, C22B4; Cell Signaling Technology, Beverly, MA, USA), p65 (for Western blotting, 3034; Cell Signaling Technology), RNF11 (described previously [20]) and V5 (MCA1360; AbD Serotec, Oxford, UK).

### Plasmids and transfections

Human RNF11 cDNA was originally subcloned into pcDNA3.1(+) (Invitrogen) using Kpn1 and Not1 restriction sites as described previously [20]. Wild-type RNF11 was cut out of pcDNA and into pFUGW with BamHI and Asc1. A V5 sequence was added to the N-terminus of the RNF11 sequence and was PCR-amplified into the plasmid. The NF-κB luciferase vector (pGL4.32[luc2P/NF-κB/Hygro]) and internal control *Renilla* vector (pGL4.74[hRluc/TK]) were purchased from Promega (Madison, WI, USA). The NF-κB luciferase vector contains a (GGGAATTTCC)_5_ NF-κB response element protein promoter. Flag-A20 was a kind gift from Dr Edward W Harhaj (Microbiology and Immunology, Miller School of Medicine, University of Miami, Miami, FL, USA). Transient transfections of SH-SY5Y and N2A cells were performed using Lipofectamine 2000 transfection reagent (Invitrogen) according to the manufacturer’s protocol.

### RNA interference

Individual siRNA duplexes were purchased from Dharmacon Inc (Chicago, IL, USA) and tested for knockdown of RNF11 using quantitative RT-PCR (qRT-PCR) in SH-SY5Y cells (not shown). The most effective sequence was cloned into pFH1UGW backbone by introducing Nhe1 and Pac1 overhangs at each end of the duplex. The sense sequence for RNF11 shRNA was 5′-GAT GAC TGG TTG ATG AGA T-3′, and the antisense sequence was 5′-ATC TCA TCA ACC AGT CAT C-3′. All constructs were verified by restriction enzyme digestion and sequencing. Lentiviruses for shRNA-RNF11 and shRNA-Scramble constructs were produced by the Emory University Viral Vector Core facility (Atlanta, GA, USA).

### Site-directed mutagenesis

Site-directed mutagenesis of RNF11 (G2A, Y40A, H119/122A or H2, I101A, C99A and silent mutations at Q72/R73 to confer shRNA resistance) was performed using the QuikChange II XL Site-Directed Mutagenesis Kit (Stratagene, Santa Clara, CA, USA) according to the manufacturer’s instructions using N-terminal V5-tagged RNF11 as a template. The primers for the myristoylation mutant (G2A) were as follows: forward, 5′-CTC GAT TCT ACG ACC GGT ATG GCG AAA TGC CTC AAA TCC CCC ACC-3′, and reverse, 5′-GGT GGG GGA TTT GAG GCA GTT CGC CAT ACC GGT CGT AGA ATC GAG-3′. The primers for the PPPY domain mutant (Y40A) were as follows: forward, 5′-GCC GCC GCC GCC AGC TCA GGA ACA AGT TCC AG-3′, and reverse, 5′-CTG GAA CTT GTT CCT GAG CTG GCG GCG GCG GC-3′. The primers for the RING domain mutant (H2) were as follows: forward, 5′-CGA TTT CTG CCG TGC ATG GCC ATC TAT GC-3′, and reverse, 5′-CTA TAC AGT CCA GGG CAT AGA TGG CCA TG-3′. The primers for the RING domain mutant (I101A) were as follows: forward 5′-GAT CCG GGA GTG TGT GGC CTG TAT GAT GGA CTT TG-3′, and reverse, 5′-CAA AGT CCA TCA TAC AGG CCA CAC ACT CCC GGA TC-3′. The primers for the RING domain mutant (C99A) were as follows: forward, 5′-GAT CCG GGA GGC TGT GAT CTG TAT GAT GGA C-3′, and reverse, 5′-GTC CAT CAT ACA GAT CAC AGC CTC CCG GAT C-3′. The primers for shRNA-RNF11 resistance were as follows: forward, 5′-CAG CTG ACT GAA GAG GAA CAA ATT AGG ATA GCT CAG AGG ATA GGT C-3′, and reverse, 5′-GAC CTA TCC TCT GAG CTA TCC TAA TTT GTT CCT CTT CAG TCA GCT G-3′. The primers were utilized for PCR amplification, which was performed according to the company’s protocol. All constructs were verified by restriction enzyme digestion and sequencing.

### TNF-α stimulation and luciferase assays

Recombinant TNF-α was purchased from R&D Systems (Minneapolis, MN, USA). All stimulations of cells with TNF-α were preceded by a 1-hour serum starvation period. Cells were stimulated with 10 ng/ml TNF-α at the indicated times. SH-SY5Y cells were transfected with the luciferase and *Renilla* plasmids for 24 hours. Cells were stimulated with TNF-α for 6 hours. Cell lysates were prepared in Passive Lysis 1× Buffer (Promega). Luciferase activity was measured using the Dual-Luciferase Reporter Assay System (Promega) according to the manufacturer’s protocol. Firefly luciferase values were normalized according to *Renilla* luciferase values. Luciferase results are presented as fold changes relative to the untreated control sample. Control samples with luciferase, *Renilla* luciferase or vector transfection were assayed to confirm the specificity of luminescence.

### Immunocytochemistry

SH-SY5Y cells were grown on coverslips coated with Matrigel (BD Biosciences, San Diego, CA, USA), and primary cortical neurons were grown on coverslips coated with poly-L-lysine (Sigma-Aldrich. After being manipulated, cells were fixed with 2% paraformaldehyde and immunostained as described previously [20] with an antibody against p65. Cell nuclei were stained with Hoechst 333258 (Molecular Probes/Invitrogen). The fluorophore-conjugated secondary antibody used was goat anti-mouse Rhodamine Red-X (Jackson ImmunoResearch Laboratories, West Grove, PA, USA). For p65 analysis, coverslips were imaged at × 40 magnification using an Olympus BX51 Fluorescence Microscope (Olympus America, Inc, Melville, NY, USA), and colocalization of p65 and 4′,6-diamidino-2-phenylindole in GFP-positive cells was quantified as described previously [33]. For each condition, at least 100 cells were quantified in the SH-SY5Y cell experiments and at least 50 cells were quantified in the primary neuron experiments. The percentage of p65 positive pixels above threshold intensity which overlapped with Hoechst staining was calculated for each cell. An area near the perimeter of the cell was used as background. For each experiment, the fold change in percentage of p65-positive pixels was normalized to unstimulated cells.

### Quantitative real-time PCR

Total RNA was isolated from cells using a standard TRIzol reagent (Invitrogen) extraction protocol. RNA was converted to cDNA using a High Capacity cDNA Reverse Transcription Kit (Ambion, Austin, TX, USA). Real-time PCR was performed with a 7500 Fast RT-PCR System (Applied Biosystems, Foster City, CA, USA) using 20 ng/μl cDNA, TaqMan Universal PCR Master Mix (Applied Biosystems) and gene-specific TaqMan probes (Applied Biosystems) against RNF11 (Hs00702517_s1, Mn00450014_m1), TNF-α (Hs00174128_m1), A20 (Hs00234712_m1), monocyte chemoattractant protein 1 (MCP-1) (Mm00441242_m1), glucuronidase-β (GUSB) (Hs99999908_m1) or glyceraldehyde-3-phosphate dehydrogenase (GAPDH) (4308313). For each RNA sample, each primer set was run in triplicate. Gene expression was normalized to the internal control GUSB or GAPDH and relative expression was calculated for each gene using the comparative cycle threshold method.

### Immunoblotting

Immunoblotting was performed as previously described [20]. Cells were transfected for 24 hours with the indicated plasmids and rinsed with PBS, then harvested in 100 μl  mM TrisHCl, pH 7.6, 150 mM NaCl, 0.1% Triton X-100, 1% Nonidet P (NP)-40, Halt Phosphatase Inhibitor Cocktail (Pierce Biotechnology, Rockford, IL, USA) and protease inhibitor cocktail (PIC) (Roche Diagnostics, Mannheim, Germany). Samples were spun at 14,000 rpm, and protein concentration was determined using the DC Protein Assay (Bio-Rad Laboratories, Hercules, CA, USA). Samples were separated by SDS-PAGE and transferred onto polyvinylidene fluoride membranes. Immunoblotting was performed using primary antibodies against A20, β-actin, Flag, histone 1, Itch, p65, RNF11 or V5. Membranes were scanned using the Odyssey Imaging System (LI-COR Biosciences, Lincoln, NE, USA). β-actin was used as a loading control where appropriate.

### Co-immunoprecipitation

Immunoprecipitation (IP) experiments were performed as described previously [34]. Equal amounts of protein were used for pull-down assays. Cell lysates were cleared with mouse immunoglobulin plus protein A Sepharose beads (Invitrogen) for 30 minutes at 4°C. IP experiments were then performed using antibodies against Flag, Itch, RNF11 or V5. Control IP experiments were performed with beads alone or with empty vector to demonstrate specificity. Immunoprecipitates were assessed by immunoblot analysis. Densitometric measurements for A20, Itch or RNF11 immunoreactivity in RNF11 immunoprecipitates were performed using ImageJ software (National Institutes of Health, Bethesda, MD, USA). Measurements were normalized to the amount of immunoreactivity of the same protein in the input samples. Unstimulated conditions were set at 100% for relative comparisons.

### ELISA

Cells were serum-starved for 1 hour prior to stimulation with 10 ng/ml TNF-α. SH-SY5Y cells were stimulated for 4 hours before being rinsed, then replaced with fresh serum-free media to eliminate detection of TNF-α, which was used to stimulate cells. Media were collected 20 hours later from SH-SY5Y cells or 24 hours after stimulation of mouse primary cortical neurons. Media were briefly centrifuged at low speed after being collected. We followed the manufacturer’s instructions for performing and analyzing the Human TNF-α ELISA Kit (Invitrogen) and the mouse MCP-1 Quantikine ELISA Kit (R&D Systems). Each sample was run in duplicate.

### Nuclear fractionation

Fractionation was performed as described previously [35]. Briefly, cells were rinsed in PBS, then harvested in cold extraction buffer (10 mM 4-(2-hydroxyethyl)-1-piperazineethanesulfonic acid, pH 7.6, 60 mM KCl, 1 mM ethylenediaminetetraacetic acid (EDTA), 0.1% NP-40, 1 mM dithiothreitol, 1 mM phenylmethylsulfonyl fluoride (PMSF), Halt Phosphatase Inhibitor Cocktail and PIC). Cells were spun at 200 *g* for 5 minutes. The supernatant was collected as the cytoplasmic fraction. The pellet was rinsed in cold extraction buffer without NP-40. The pellet was resuspended in nuclear extraction buffer (20 mM Tris, pH 8.0, 420 mM NaCl, 1.5 mM MgCl_2_, 0.2 mM EDTA, 0.5 mM PMSF, 25% glycerol, Halt Phosphatase Inhibitor Cocktail and PIC). The final salt concentration was adjusted to 400 mM before lysates were kept on ice for 10 minutes with occasional agitation. Lysates were spun at 16,000 *g* for 10 minutes, and the supernatant was collected as the nuclear fraction. Fractions were examined using immunoblot analysis. Densitometric measurements for actin, histone 1 and p65 immunoreactivity in cytoplasmic and nuclear fractions were performed using ImageJ software. p65 measurements were normalized to the amount of immunoreactivity of the loading control. Unstimulated conditions were set at 100% to make relative comparisons.

### Statistical analysis

Statistical analysis was performed using GraphPad Prism version 4.03 software (GraphPad Software, Inc, La Jolla, CA, USA). One-way analysis of variance (ANOVA) was performed for analysis of qRT-PCR for RNF11 levels and luciferase assays. Tukey’s posttest was used for the luciferase assay with mutant RNF11 constructs, and Bonferroni posttests were used for the remaining ANOVAs. Two-way ANOVAs with repeated measures were performed for analysis of p65 translocation, and standard two-way ANOVA was used to analyze the mRNA and protein expression of inflammatory markers and the densitometric analysis of p65 fractions. Bonferroni posttests were performed for all two-way ANOVAs. Densitometric analysis of co-IPs was performed using paired *t*-tests. Statistical significance was set at *P* < 0.05. All experimental results are presented as means ± SEM for at least three independent experiments.

## Results

### RNF11 modulates TNF-α-induced neuronal NF-κB activation

**Figure 2:**
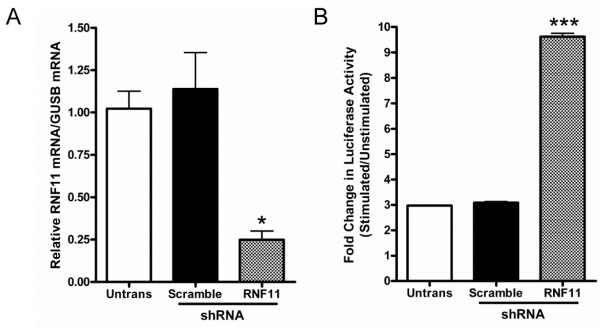
**Increased NF-κB luciferase activity in neuronal cells with knockdown of RNF11.(A)** Stable SH-SY5Y cell lines were generated using lentiviruses containing a control scramble small hairpin RNA (shRNA) sequence (shRNA-Scramble cells) or shRNA targeted against RNF11 (shRNA-RNF11 cells). Quantitative RT-PCR was used to measure relative RNF11 mRNA levels with glucuronidase β as an internal control. Values are expressed as mean comparative cycle threshold ± SEM of triplicate experiments. **(B)** Stable SH-SY5Y cell lines, as well as untransduced cells, were transiently cotransfected with luciferase and *Renilla* plasmids and stimulated with 10 ng/ml TNF-α or PBS for 6 hours. Values represent the fold change in NF-κB-dependent activity as measured by the ratio of luciferase to *Renilla* luminescence in stimulated over unstimulated samples ± SEM in triplicate experiments. ^*^P < 0.05; ^***^P < 0.001.

To examine the effects of RNF11 on neuronal NF-κB signaling, we transduced SH-SY5Y cells with lentivirus expressing shRNA targeted against RNF11 (shRNA-RNF11 cells) or scramble shRNA sequence (shRNA-Scramble cells). qRT-PCR was used to measure the efficiency of RNF11 knockdown. The endogenous RNF11 mRNA level was reduced by 75% in shRNA-RNF11 cells compared to untransduced cells (*P* < 0.05) and shRNA-Scramble cells (*P* < 0.05) (Figure 2A). To measure the activity of NF-κB signaling in these cell lines, shRNA-RNF11 cells and shRNA-Scramble cells were transiently cotransfected with plasmids containing an NF-κB response element driving expression of firefly luciferase or the T7 promoter driving expression of *Renilla* luciferase. TNF-α specifically binds and cross-links TNFR-1 and TNFR-2, which are members of the TNFR superfamily and are constitutively expressed in the brain; moreover, binding of TNF-α to its receptors causes downstream activation of NF-κB signaling in neurons [36]. Cells were exposed to 0 or 10 ng/ml of TNF-α for 6 hours. NF-κB-dependent firefly luciferase activity levels were normalized to control *Renilla* luciferase activity, and the level of NF-κB activity was calculated as the fold change between stimulated and unstimulated samples. Stimulation with TNF-α caused a 10-fold increase in NF-κB activity in the shRNA-RNF11 cells, which was significantly different from the observed 3-fold increase in NF-κB activity in untransduced cells (*P* < 0.001) and shRNA-Scramble cells (*P* < 0.001) (Figure 2B). No difference was observed between shRNA-Scramble cells and untransduced cells. These results indicate that reduced expression of RNF11 increases NF-κB activation in neuroblastoma cells.

**Figure 3:**
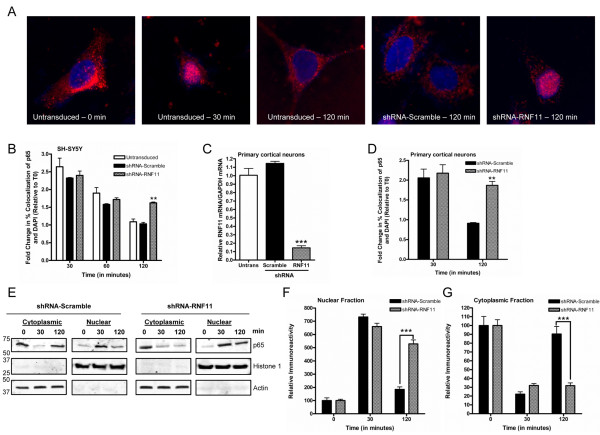
**Prolonged nuclear p65 translocation after TNF-α stimulation observed in neuronal cells with knockdown of RNF11.(A)** SH-SY5Y cell lines (untransduced, small hairpin RNA (shRNA)-RNF11, shRNA-Scramble) were stimulated with TNF-α for 0, 30, 60 or 120 minutes. Cells were immunostained for p65 (red) and nuclear DNA (Hoechst 333258, blue). Representative images obtained at 0, 30 and 120 minutes after stimulation are shown. **(B)** Cells were analyzed for overlap of p65-positive and Hoechst 333258-positive pixels. The fold change in percentage overlap of p65- and Hoechst 333258-positive pixels was calculated relative to unstimulated conditions. **(C)** Murine primary cortical neurons were treated with cytosine arabinoside and transduced with lentiviruses containing shRNA targeted against RNF11 (shRNA-RNF11 neurons) or scramble shRNA sequence (shRNA-Scramble neurons). Quantitative RT-PCR (qRT-PCR) was used to measure relative RNF11 mRNA levels. **(D)** shRNA-RNF11 or shRNA-Scramble neurons were stimulated for 0, 30 or 120 min before being immunostained for p65 and Hoechst 333258. Transduced cells were analyzed for overlap of p65- and Hoechst 333258-positive cells. The fold change in percentage overlap of p65- and Hoechst 333258-positive pixels was calculated relative to unstimulated conditions. **(E)** shRNA-Scramble and shRNA-RNF11 cells were stimulated for 0, 30 or 120 minutes. Cytoplasmic and nuclear fractions were resolved by SDS-PAGE. **(F)** ImageJ software was used to quantify the ratio of the densitometry of the p65 bands in the nuclear fractions to the density of histone 1 immunoreactivity. The ratio for each time point was compared relative to the steady-state ratio, which was set at 100%. **(G)** ImageJ software was used to quantify the ratio of the densitometry of the p65 bands in the cytoplasmic fractions in a manner similar to that described in (F). All p65 colocalization values are means ± SEM of triplicate experiments. qRT-PCR values are expressed as mean comparative cycle threshold ± SEM of triplicate experiments. Western blots of cytoplasmic and nuclear fractions were run from triplicate experiments. ^**^P < 0.01; ^***^P < 0.001.

Canonical NF-κB signaling involves phosphorylation of IκBα by the IκB kinase (IKK) complex as a result of upstream receptor and associated complex activation. IκBα is a cytoplasmic protein that binds NF-κB transcription factors, including p65, to inhibit their activity. Phosphorylation of IκBα releases p65 and other transcription factors from the complex and allows them to translocate to the nucleus and drive NF-κB-dependent transcription [37]. To investigate whether changes in RNF11 expression affect p65 translocation to the nucleus, we immunostained shRNA-RNF11 and shRNA-Scramble cells with p65 and Hoechst 333258, a nuclear stain, and analyzed them for p65 colocalization with Hoechst 333258 following stimulation with TNF-α for 0, 30, 60 or 120 minutes. The fold change in colocalization at steady state for each cell line was calculated. After 30 minutes of stimulation, an increase in p65 immunoreactivity in the nucleus was observed in all cell lines. Representative images are shown in Figure 3A, and quantification is shown in Figure 3B. p65 overlap with Hoechst 333258 decreased at 60 minutes, as well as at 120 minutes, to the levels observed at 0 minutes. The overlap between p65 and Hoechst 333258 in both untransduced and shRNA-Scramble cells was similar to levels observed at 0 minutes. Conversely, the amount of p65 overlap with Hoechst 333258 at 120 minutes was nearly 60% higher in shRNA-RNF11 cells than in untransduced cells (*P* < 0.01) or shRNA-Scramble cells (*P* < 0.01). No significant differences between untransduced and shRNA-Scramble cells were observed at any time point.

To validate RNF11’s effects on NF-κB signaling in primary cells, analyses for p65 translocation were performed in murine cortical neurons. Neurons were transduced with lentivirus driving expression of shRNA targeted against RNF11 or a scramble shRNA sequence, and qRT-PCR analyses revealed approximately 85% knockdown of endogenous RNF11 in shRNA-RNF11 neurons compared to untransduced neurons (*P* < 0.001) and shRNA-Scramble neurons (*P* < 0.001) (Figure 3C). No significant difference in RNF11 expression was observed between untransduced and shRNA-Scramble neurons. Neurons were analyzed for colocalization of p65 and Hoechst 333258 following stimulation with TNF-α for 0, 30 and 120 minutes. After 30 minutes of TNF-α stimulation, an approximately twofold increase in p65 immunoreactivity in the nucleus was observed under both conditions (Figure 3D). At 120 minutes poststimulation, p65 overlap with Hoechst 333258 in shRNA-Scramble cells was similar to levels observed at 0 minutes, whereas colocalization between p65 and Hoechst 333258 remained elevated in shRNA-RNF11 cells (*P* < 0.01) (Figure 3D). The results of these experiments reveal that reduced RNF11 expression leads to persistent p65 localization to the nucleus. Coupled with the NF-κB reporter analyses described above, these results strongly suggest that RNF11 is a negative regulator of neuronal NF-κB signaling.

To support the p65 immunofluorescence data, SDS-PAGE was performed to examine p65 levels in cytoplasmic and nuclear fractions from SH-SY5Y shRNA-Scramble and shRNA-RNF11 cells that were isolated after 0, 30 or 120 minutes of TNF-α stimulation. Western blot analyses revealed that p65 immunoreactivity decreased in the cytoplasmic fraction by 78% but increased more than sevenfold in the nuclear fraction after 30 minutes of TNF-α stimulation in shRNA-Scramble cells (see Figure 3E and quantification in Figures 3F and 2G). After 120 minutes of TNF-α exposure, p65 immunoreactivity decreased in the nuclear fraction and increased in the cytoplasmic fraction to approximately steady-state levels in the shRNA-Scramble samples. In contrast, levels of p65 immunoreactivity from shRNA-RNF11 samples after 30 and 120 minutes of stimulation in the cytoplasmic and nuclear fractions did not significantly change. We observed an approximately 70% decrease from steady state at both time points in the cytoplasmic fractions and more than 6.5-fold and 5-fold increases at 30 and 120 minutes, respectively, from steady state in the nuclear fractions (*P* < 0.001). These results are consistent with our hypothesis that RNF11 is a negative regulator of TNF-α-induced canonical NF-κB signaling in neurons.

### Neuronal RNF11 dynamically associates with the A20-ubiquitin editing protein complex

**Figure 4:**
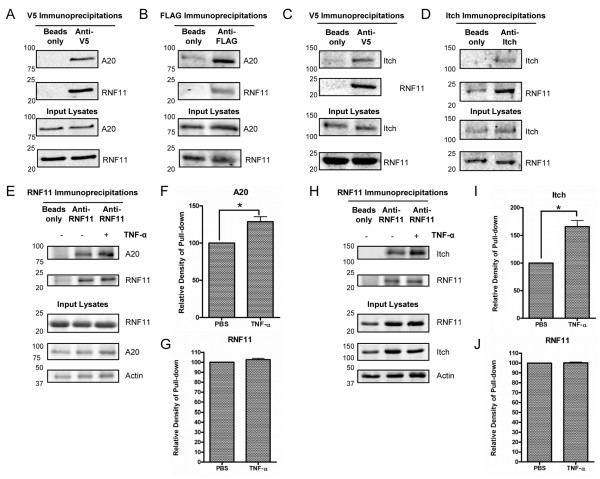
**Dynamic associations of RNF11 with both A20 and Itch in primary neurons.** N2A cells transduced with V5-RNF11 lentivirus (N2A V5-RNF11) and transfected with FLAG-A20 were harvested for immunoprecipitation (IP) with V5 antibody **(A)** or harvested for IP with FLAG antibody **(B)**. Proteins were resolved by SDS-PAGE and immunoblotted with anti-A20 and RNF11. In parallel, pull-down assays with V5 antibody **(C)** or with Itch antibody **(D)** from N2A V5-RNF11 cell lysates were resolved by SDS-PAGE. Immunoprecipitates and lysates were immunoblotted with anti-Itch and RNF11. **(E)** and **(H)** Murine primary cortical neurons were stimulated with 10 ng/ml TNF-α for 0 or 30 minutes and harvested for IP with RNF11 antibody. Control IP experiments were performed with antibody omitted. Proteins were resolved by SDS-PAGE and immunoblotted with anti-A20, Itch, RNF11 and actin. **(F)**, **(G)**, **(I)** and **(J)** ImageJ software was used to quantify the densitometry of the immunoprecipitated bands relative to the 0-minutes time point. Each input sample’s immunoreactivity was used as a loading control. All IPs are representative of at least three independent experiments. ^*^P < 0.05.

The results of previous studies in a monocyte cell line suggest that united members of the A20 ubiquitin-editing protein complex are required for mitigation of the canonical NF-κB signaling pathway [38]. The essential members of the complex are the deubiquitinase and E3 ligase A20, the E3 ligase Itch, TAX1BP1 and RNF11 [16,19]. The A20 ubiquitin-editing protein complex is responsible for deubiquination of K63 ubiquitin linkage on receptor interacting protein 1 (RIP1) and attachment of K48 ubiquitin linkage to RIP1, which thereby facilitates proteasomal degradation of RIP1 and termination of NF-κB signaling [37]. To determine whether RNF11 associates with the A20 ubiquitin-editing protein complex in a neuronal system, we performed coimmunoprecipitation (co-IP) experiments in N2A cells. Because of low endogenous expression of RNF11, N2A cells were transduced with lentivirus expressing wild-type RNF11 tagged with a V5 epitope (N2A V5-RNF11). Additionally, owing to the low level of endogenous A20, N2A V5-RNF11 cells were transiently transfected with plasmid expressing FLAG-tagged A20 (FLAG-A20). V5-RNF11 immunoprecipitates were enriched with A20 immunoreactivity, and control immunoprecipitates with V5 antibody omitted were absent of A20 (Figure 4A). Reciprocal co-IPs revealed that RNF11 was enriched in FLAG pull-down assays for FLAG-A20 (Figure 4B), indicating that RNF11 and A20 associate in neuroblastoma cells. In parallel, co-IPs were performed to examine RNF11’s association with Itch. Pull-down assays with V5 antibody from N2A V5-RNF11 cell lysates were enriched with Itch immunoreactivity, whereas control co-IPs with V5 antibody omitted were absent for Itch (Figure 4C). Reciprocal co-IPs showed V5-RNF11 enrichment in endogenous Itch immunoprecipitates (Figure 4D). These findings indicate that RNF11 associates with A20 and Itch in neuronal cell lines and suggests that at least a portion of the A20 ubiquitin-editing protein complex exists in neuronal systems.

To determine whether TNF-α stimulation affects RNF11’s association with the A20 ubiquitin-editing protein complex, co-IP experiments were performed in primary neurons following stimulation with TNF-α for 0 or 30 minutes. RNF11 immunoprecipitates were enriched with A20 immunoreactivity at steady state and following TNF-α stimulation (Figure 4E), and a significant increase in A20 immunoreactivity was observed following TNF-α stimulation for 30 minutes in RNF11 immunoprecipitates (0 minutes: 100%, 30 minutes: 128.7%; *P* < 0.05) (Figure 4F). Additionally, parallel co-IP experiments were conducted to examine the association between RNF11 and Itch following TNF-α stimulation. RNF11 immunoprecipitates were enriched with Itch immunoreactivity under both steady-state and TNF-α stimulation conditions (Figure 4H), and, like A20, a significant increase in Itch immunoreactivity was observed in RNF11 immunoprecipitates following stimulation with TNF-α (0 minutes: 100%, 30 minutes: 165.8%; *P* < 0.05) (Figure 4I). Importantly, RNF11 levels were similar for all immunoprecipitates (see Figures 4E and 3H, with quantification shown in Figures 4G and 3J). These results demonstrate that RNF11 can exist in complexes with A20 and Itch in neurons and that TNF-α stimulation enhances the association of A20 and Itch with RNF11.

### The myristoylation domain is necessary for RNF11-mediated inhibition of NF-κB signaling

**Figure 5:**
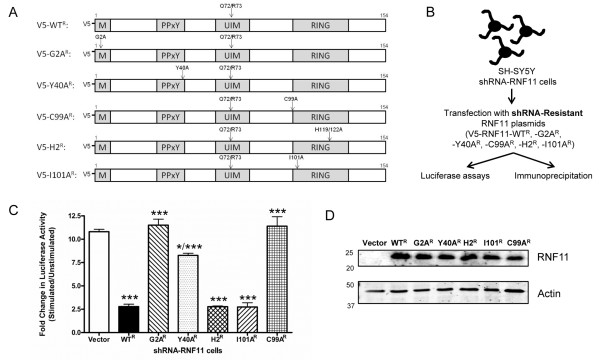
**Myristoylation mutant of RNF11 is unable to alter NF-κB signaling.(A)** Site-directed mutagenesis of V5-tagged wild-type RNF11 was used to create the myristoylation mutant (G2A), the PPxY motif mutant (Y40A) and RING domain mutants (C99A, H2, I101A). Small hairpin (shRNA)-resistant mutants were created using Q72/R73 mutagenesis. The domains were as follows: M, myristoylation domain; PPxY, PPxY motif; UIM, ubiquitin-interacting motif; and RING, RING-H2 domain. **(B)** Model of resistance experiments in SH-SY5Y cells. Stably transduced shRNA-RNF11 cells were transfected with shRNA-resistant RNF11 plasmids to determine which mutants behaved similarly to the endogenous protein in luciferase and coimmunoprecipitation experiments. **(C)** SH-SY5Y shRNA-RNF11 cells were transfected with shRNA-resistant RNF11 mutants and luciferase and *Renilla* constructs. Cells were exposed to 10 ng/ml TNF-α for 6 hours and subjected to a luciferase assay. Values represent the fold changes in the ratios ± SEM of luciferase to *Renilla* luminescence of stimulated over unstimulated samples of triplicate samples in three experiments. **(D)** Samples from (C) were resolved by SDS-PAGE and immunoblotted with RNF11 antibody. Blots are representative of three independent experiments. ^*^P < 0.05; ^***^P < 0.001.

The myristoylation domain of RNF11 is necessary for proper recruitment to early endosomes, whereas the PPxY motif is important for RNF11’s associations with Itch-, Smurf2- and Nedd4-like proteins [23,39]. The RING domain of RNF11 is important for interactions with proteins involving ubiquitination and degradation [22]. Although the substrate for RNF11’s putative E3 ubiquitin ligase activity has yet to be reported, researchers in previous studies have identified mutations that interfere with the ubiquitination activity of the RING domain in analogous E3 ubiquitin ligases [40]. Mutations of cysteines or histidines within the RING domain cause structural changes that prevent E2 binding and ubiquitination, whereas an isoleucine mutation within the RING domain prevents interactions with E2 enzymes but keeps the RING domain structure intact [41]. We generated RNF11 mutants for each of these domains to examine their importance with respect to RNF11’s influence over neuronal NF-κB signaling (Figure 5A). A myristoylation mutant was made by substituting alanine for glycine 2 (RNF11-G2A) [39], and a PPxY motif mutant was constructed by substituting alanine for tyrosine 40 to abolish binding with Nedd4-like proteins (RNF11-Y40A) [23,39]. RING domain mutants were generated by substituting alanine for isoleucine 101 to prevent E2 binding (RNF11-I101A) [41], or by substituting alanine for histidine 119 and histidine 122 to abrogate RING domain structure (RNF11-H2) [40], or by substituting alanine for cysteine 99 to abolish proper RING domain folding (RNF11-C99A) [40]. Importantly, additional site-directed mutagenesis was performed to generate shRNA-resistant forms of these RNF11 mutants. This approach allowed for the expression of RNF11 mutants under reduced expression of endogenous RNF11 in NF-κB-dependent luciferase assays and co-IPs (Figure 5B). Silent mutations were introduced at glutamine 72 and arginine 73 [19] in wild-type RNF11, the myristoylation mutant, the PPxY motif mutant and the three RING domain mutants to generate the following shRNA-resistant RNF11 constructs: V5-WT^R^, V5-G2A^R^, V5-Y40A^R^, V5-I101A^R^, V5-H2^R^ and V5-C99A^R^, respectively (Figure 5A).

To determine the impact of RNF11’s functional amino acid motifs with respect to NF-κB signaling, we conducted the luciferase assays described in Figure 2B. SH-SY5Y shRNA-RNF11 cells were transiently cotransfected with empty vector, V5-WT^R^, V5-G2A^R^, V5-Y40A^R^, V5-I101A^R^, V5-H2^R^ or V5-C99A^R^ constructs, as well as plasmids containing an NF-κB response element driving expression of firefly luciferase or the T7 promoter driving expression of *Renilla* luciferase. Cells were exposed to 0 or 10 ng/ml of TNF-α, and luciferase readouts were measured after 6 hours of TNF-α stimulation. Expression of V5-WT^R^, V5-H2^R^ or V5-I101A^R^ in shRNA-RNF11 cells decreased NF-κB activity by approximately 75% compared to vector-transfected cells (*P* < 0.001) (Figure 5C). Transfection of V5-G2A^R^ or V5-C99A^R^ did not significantly alter NF-κB activity in comparison to vector transfected cells, but was significantly different from cells transfected with V5-WT^R^ (*P* < 0.001). Last, expression of V5-Y40A^R^ reduced NF-κB activity only 24%, a significant difference from both vector and V5-WT^R^ (*P* < 0.05 for vector and V5-Y40A^R^; *P* < 0.001 for V5-WT^R^ and V5-Y40A^R^). Western blot analyses indicated similar expression levels of RNF11 mutants (Figure 5D). These results suggest that the myristoylation domain is required and the PPxY motif is necessary for the full effect of RNF11’s influence over NF-κB activation as measured by luciferase assay. Moreover, cysteine 99 is required, but isoleucine 101 or histidines 199 and 122 are dispensable, for RNF11-mediated suppression of NF-κB activity under these experimental conditions.

**Figure 6:**
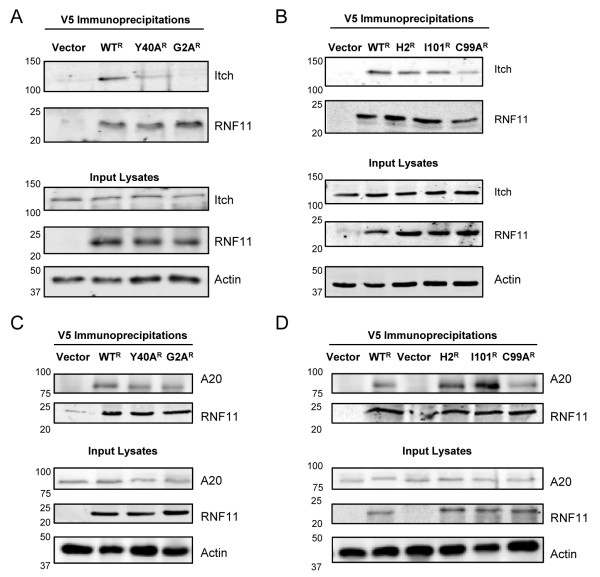
**Myristoylation mutant of RNF11 is unable to associate with Itch.** SH-SY5Y shRNA-RNF11 cells were transfected with shRNA-resistant RNF11 constructs or vector. Coimmunoprecipitation experiments using V5 antibody were performed 24 hours after transfection. Immunoprecipitates and lysates were resolved by SDS-PAGE and immunoblotted with anti-A20, Itch, RNF11 or actin. Blots are representative of three independent experiments.

To determine whether mutations in the myristoylation domain, PPxY motif or RING domain alter RNF11’s association with the A20 ubiquitin-editing protein complex, SH-SY5Y shRNA-RNF11 cells were transfected with vector, V5-WT^R^, V5-G2A^R^, V5-Y40A^R^, V5-I101A^R^, V5-H2^R^ or V5-C99A^R^, and co-IPs for endogenous Itch were performed. V5-WT^R^, V5-I101A^R^, V5-H2^R^ and V5-C99A^R^ immunoprecipitates were enriched with Itch immunoreactivity compared to vector control (Figures 6A and 5B), whereas V5-G2A^R^ immunoprecipitates were absent for Itch (Figure 6A). V5-Y40A^R^ immunoprecipitates displayed endogenous Itch immunoreactivity, although it was reduced compared to V5-WT^R^ immunoprecipitates (Figure 6A). Additionally, SH-SY5Y shRNA-RNF11 cells were transfected with vector, V5-WT^R^, V5-G2A^R^, V5-Y40A^R^, V5-I101A^R^, V5-H2^R^ or V5-C99A^R^, as well as FLAG-A20, and co-IPs for A20 were performed. The immunoprecipitate of each RNF11 construct tested was enriched with A20 immunoreactivity compared to vector control (Figure 6C and 6D). These studies revealed that the myristoylation domain is necessary for RNF11 association with Itch and that the PPxY motif is also important. On the basis of these results and the luciferase assays described above, we hypothesize that disrupted RNF11-Itch complex formation contributes to the impaired regulation of NF-κB activation exhibited by the RNF11 myristoylation domain and PPxY motif mutants.

### RNF11 influences inflammatory responses in neurons

Inflammation is tightly associated with NF-κB signaling as well as with neurological disease; therefore, we investigated whether RNF11 is linked to inflammatory response induction. To test this hypothesis, SH-SY5Y shRNA-RNF11 and shRNA-Scramble cells were exposed to 0 or 10 ng/ml TNF-α for 4 hours, and RNA was extracted to examine by qRT-PCR the induction of inflammatory cascades. Specifically, mRNA levels of the proinflammatory cytokine TNF-α and the cytokine-inducible protein A20 were measured [42-45]. TNF-α is transcriptionally regulated by NF-κB as well as by other inflammation-related transcription factors; therefore, increased TNF-α mRNA levels are generally indicative of an immune response involving multiple signaling pathways [46]. Changes in A20 mRNA levels are linked more specifically to activity of NF-κB signaling [47,48]. TNF-α stimulation increased A20 mRNA levels in shRNA-Scramble cells; however, a more substantial increase in A20 mRNA levels was observed in shRNA-RNF11 cells (shRNA-Scramble: 3.81-fold change, shRNA-RNF11: 38.70-fold change; *P* < 0.001) (Figure 1A). Similarly, TNF-α stimulation increased TNF-α mRNA levels in shRNA-Scramble cells, but to a greater degree in shRNA-RNF11 cells (shRNA-Scramble: 3.08-fold change, shRNA-RNF11: 9.70-fold change; *P* < 0.001) (Figure 1B). Importantly, steady-state levels of A20 and TNF-α mRNA were not significantly different between shRNA-RNF11 cells and shRNA-Scramble cells. To further support these findings, we performed a TNF-α ELISA to measure secreted TNF-α protein levels. After serum starvation for 1 hour, cells were stimulated with 0 or 10 ng/ml TNF-α for 4 hours. To minimize detection of the human recombinant TNF-α used to stimulate the cells, cells were rinsed with serum-free media and incubated with a reduced volume of serum-free media for 20 hours. Basal levels of TNF-α were similar in shRNA-Scramble (10.49 pg/ml) and shRNA-RNF11 cells (below the limits of detection) (Figure 1C). Recombinant TNF-α stimulation of the NF-κB pathway increased production of TNF-α protein in shRNA-Scramble cells and, to a larger extent, in shRNA-RNF11 cells (shRNA-Scramble: 744.51, shRNA-RNF11: 1,285.03 pg/ml; *P* < 0.001).

To expand these studies to primary cells, murine cortical neurons were transduced with lentivirus expressing shRNA-Scramble or shRNA-RNF11 and MCP-1 mRNA levels were measured. MCP-1 is a potent chemokine that attracts monocytes, macrophages and microglia to sites of injury to evoke further release of inflammatory molecules, and it is involved in inflammatory neuropathologies [47,49-51]. Importantly, like TNF-α and A20, MCP-1 is transcriptionally regulated by NF-κB [52,53]. In our primary neuron-enriched cultures, we specifically examined effects of reduction of the high RNF11 mRNA expression in neurons (in comparison to glial cells, as shown in Figure 1D) due to the cytosine arabinoside treatment of our cultures. Stimulation with 10 ng/ml TNF-α revealed a time-dependent increase in MCP-1 mRNA levels at 4 and 24 hours poststimulation in shRNA-Scramble neurons, whereas a greater increase was observed at each time point in neurons transduced with shRNA-RNF11 (shRNA-Scramble 0 hours: 1.02-fold change, 4 hours: 6.38-fold change, 24 hours: 14.91-fold change; shRNA-RNF11: 0 hours: 1.81-fold change, 4 hours: 25.73-fold change, 24 hours: 29.04-fold change; *P* < 0.05 for both time and transduction differences) (Figure 1E). This result was confirmed by analyzing MCP-1 protein levels from conditioned media of stimulated cells. Transduced shRNA-Scramble or shRNA-RNF11 primary cortical neurons were stimulated with 0 or 10 ng/ml TNF-α in a reduced volume of serum-free media for 24 hours. Basal levels of MCP-1 were similar in media collected from shRNA-Scramble (9.41 pg/ml) and shRNA-RNF11 cells (9.53 pg/ml). TNF-α stimulation increased MCP-1 protein levels in media collected from shRNA-Scramble cells and, to a greater degree, from shRNA-RNF11 cells (shRNA-Scramble: 1,976.60, shRNA-RNF11: 3,654.02 pg/ml; *P* < 0.001) (Figure 1F). These results suggest that changes in RNF11 expression influence mRNA and protein levels of gene products that are downstream targets of NF-κB signaling. Therefore, we propose that RNF11 likely contributes to the mitigation of inflammatory responses exerted by neurons.

## Discussion

NF-κB activity is essential for peripheral immune cell survival, and proper regulation of NF-κB signaling is critical for mounting a normal immune response [54]. Persistent activation of the NF-κB pathway is known to promote inflammation and inflammatory diseases [54,55], including progressive neurodegenerative diseases [2-11]. Notably, inhibition of NF-κB activity has been demonstrated to attenuate neuroinflammation and cell death in animal models of PD, Alzheimer disease (AD) and ischemia [5,8,9,56,57]. In this study, we investigated the role of neuronal RNF11 as a component of the A20 ubiquitin-editing complex in regulating TNF-α-induced canonical NF-κB activity by targeted knockdown of endogenous RNF11 in human neuroblastoma cells and primary neuronal cultures. Our analyses revealed that (1) neuronal RNF11 acts as a negative regulator of the canonical NF-κB signaling pathway, (2) neuronal RNF11 associates with the A20 ubiquitin-editing protein complex, (3) the myristoylation and PPxY domains of RNF11 are required and necessary, respectively, for RNF11’s effects on NF-κB signaling and association with Itch, a member of the A20-ubiquitin editing protein complex and (4) reduced expression of RNF11 in neurons can result in aberrant regulation of inflammatory signaling. Together these findings suggest that RNF11 has a critical role in the regulation of canonical NF-κB signaling in the central nervous system.

NF-κB responds to a diverse series of inflammatory activators, including ultraviolet light, double-stranded RNA, cytokines, vasoactive peptides and viral oncogenes [58]. Stimulation of the NF-κB signaling pathway causes transcription of various inflammatory markers, including inducible chemokines, cell adhesion molecules and vasoactive and antiapoptotic proteins important in the cellular stress response to effective control of inflammation [59]. The signaling pathway also has several levels of autoregulation, one of which includes the activation and association of the RIP1 and IκB kinases by the A20 ubiquitin-editing protein complex [59]. Through the degradation of RIP1 and the disruption of the association between RIP1 and IKK, the A20 ubiquitin-editing protein complex efficiently halts canonical NF-κB signaling [59]. Outside the nervous system this complex requires the association of A20, Itch, TAX1BP1 and RNF11 to enhance K48 ubiquitination of RIP1 and promote RIP1 targeting to the proteasome [16,19]. Recently, a human genomewide siRNA screen identified RNF11 as an important modulator of canonical NF-κB activity in HEK-293 cells [60]. In this report, we have demonstrated that reduction of RNF11 in primary cortical neurons sustained canonical NF-κB signaling, most likely by RNF11’s lack of association with the A20 ubiquitin-editing protein complex. A yeast two-hybrid screen revealed that Itch, TAX1BP1, NF-κB essential modulator and A20 are potential binding partners of RNF11 [25]. Other researchers have reported associations of RNF11 with *glutathione S-transferase* -tagged Itch in breast cancer cell lines [21], tagged RNF11 with tagged TAX1BP1 and A20 in HEK cells [19] and RNF11 with A20, TAX1BP1 and RIP1 in mouse embryonic fibroblasts and blood-derived macrophages [19]. Our present work reveals that RNF11 associates with Itch and A20 in neuronal systems and that there is an increased association of A20 and Itch with RNF11 after TNF-α stimulation (Figure 4). This phenomenon may be due to enhanced stability of the A20 ubiquitin-editing protein complex through protein changes such as posttranslational modifications. Importantly, our data suggest that neurons, similarly to monocytic cells [19], form an A20 ubiquitin-editing protein complex to garnish transient reactions for regulating the canonical NF-κB signaling pathway.

Our studies involving RNF11 mutants indicate that the myristoylation domain is imperative for RNF11’s effects on NF-κB activity as well as for association with the A20 ubiquitin-editing protein complex (Figures 5 and 6). Mutagenesis of glycine 2 disrupts the protein’s ability to incorporate myristoylic acid, causing a shift in localization from cytosolic vesicle-like structures to a more diffuse pattern throughout the cell [39]. Furthermore, mutation of RNF11’s glycine 2 prevents association and ubiquitination by Itch and Nedd4 [39]. V5-G2A^R^ most likely does not localize to intracellular compartments, where it interacts with other members of the A20 ubiquitin-editing protein complex and therefore fails to reduce NF-κB-induced luciferase activity and co-IP with Itch (Figures 5C6A and 6 C).

RNF11 also harbors a PPxY motif that previously has been shown to be important for associations with Itch and other Nedd4-like proteins [19,21,39,61]. In contrast, we have shown in this study that V5-Y40A^R^ associated with endogenous Itch in neuroblastoma cells, although Itch immunoreactivity was reduced compared to wild-type RNF11. Santonico *et al*. proposed that RNF11 may have an alternative binding site (VPxY) for Itch and structurally similar proteins [39], which may explain why we observed some degree of Itch and RNF11 interaction. Santonico *et al*. believe that this nearby site explains why some degree of RNF11 ubiquitination by Itch occurs when the consensus PPxY site is mutated. It would be interesting to explore whether this site is important for interactions with Itch or how a double-PPxY and VPxY mutation would affect RNF11 association with the A20 complex. Additionally, we observed some reconstitution of negative regulation of NF-κB signaling following expression of V5-Y40A^R^, whereas Shembade *et al*. did not [19]. This contrast may be attributed to experimental differences in measuring NF-κB signaling. Shembade *et al*. used Western blot analysis to examine transient phosphorylation of IκBα and JNK, whereas we employed quantitative luciferase assays.

The three RNF11 constructs we generated harboring RING domain mutations all maintained interactions with Itch and A20 (Figure 6B and 6D). Previously, Shembade *et al*. showed that RNF11 with a mutation of cysteine 99 to alanine was unable to associate with A20, TAX1BP1 or RIP1 in TNF-α-stimulated THP-1 cells [19]. Similarly to our studies, Santonico *et al*. found that an RNF11 RING domain mutant with serines substituted at cysteines 99 and 102 retained binding to Itch in HEK cells [39]. This discrepancy may underlie a putative difference between innate immune cells and other human cell lines in the stability and transient nature of the associations among A20-ubiquitin protein complex members.

The RING domain mutants we examined altered NF-κB-dependent luciferase activity differently, despite maintaining associations with Itch and A20. Although the mutation of isoleucine 101 and the mutations of histidines 119 and 122 recapitulated effects of wild-type RNF11, the mutation of cysteine 99 did not mimic wild-type RNF11 in this assay. Similarly, Shembade *et al*. showed that mutation of cysteine 99 to alanine was unable to restore negative regulation of NF-κB signaling in siRNA rescue experiments [19]. The results of our study support these data and add quantitative assessment of luciferase-dependent NF-κB activity of this mutant (Figure 5C). However, the incongruity between the three RING domain mutants in the NF-κB-dependent luciferase assay highlights an essential remaining question: Is RNF11’s E3 ubiquitin ligase activity critical for negative regulation of NF-κB signaling by the A20 complex?

NF-κB family proteins and the A20 ubiquitin-editing protein complex, though differentially distributed in various cells, are highly expressed in peripheral immune cells, including lymphocytes and macrophages [38,54,62-64]. RNF11 mRNA and protein levels are higher in neurons than in glial cells, including microglia (Figure 1D). The reason for this differential expression in neurons is not clear, but could be a protective strategy in postmitotic cells which have a limited lifespan and limited tolerance to chronic NF-κB activation and neuroinflammation [19]. Our studies show that reduced levels of RNF11 in neurons increase production of the inflammatory cytokine MCP-1 following TNF-α activation (Figure 1). Other agents targeting NF-κB signaling (that is, Nurr1, α-synuclein, amyloid-β) have been shown to increase production of inflammatory cytokines [10,65-68]. In neurodegenerative disease and ischemic attack, it is hypothesized that signals sent from damaged neurons and glial cells cause activation of NF-κB signaling [69]. Chronic stimulation of the NF-κB signaling cascade brings about increased production of inflammatory molecules such as TNF-α, nitric oxide and IL-1, resulting in oxidative stress and neuronal degeneration [70-72]. This can be observed in the brains of patients with stroke, AD, PD, ALS and other neurodegenerative diseases [7,73-77], and it has been hypothesized that the pathogenesis and progression of neurological diseases is in part due to dysregulation or chronic activation of the NF-κB pathway [6,78,79]. Notably, RNF11 is a candidate gene for PD [80] and colocalizes with α-synuclein-positive Lewy bodies and neurites in PD patients [20]. Our future studies will focus on investigating the role of RNF11 as a regulator of neuroinflammation in animal models of neurodegenerative diseases.

## Conclusions

Given the mounting evidence that neuroinflammation is heightened in neurodegenerative diseases [5,8,9,56,81], we conclude that functionally compromised or reduced expression of RNF11 could result in persistent NF-κB activation and could promote chronic neuroinflammation. Further studies will explore these hypotheses in relevant animal models to ascertain how RNF11 expression levels affect NF-κB signaling *in vivo*. Additionally, the impact of posttranslational modifications on RNF11 activity will be explored to gain further understanding of RNF11’s role within the A20 ubiquitin-editing protein complex in neurons.

## Abbreviations

co-IP: coimmunoprecipitation; DMEM: Dulbecco’s modified Eagle’s medium; ELISA: enzyme-linked immunosorbent assay; FCS: fetal calf serum; GFP: green fluorescent protein; IκB: inhibitor of nuclear factor κB; IKK: inhibitor of nuclear factor κB kinase; IL: interleukin; IP: immunoprecipitation; MCP-1: monocyte chemoattractant protein 1; NF-κB: nuclear factor κ light-chain enhancer of activated B cells; PBS: phosphate-buffered saline; PCR: polymerase chain reaction; qRT-PCR: quantitative real-time polymerase chain reaction; RING: really interesting new gene; RNF11: RING finger protein 11; RIP1: receptor-interacting protein 1; siRNA: small interfering RNA; Smad4: SMAD family member 4; Smurf2: SMAD-specific E3 ubiquitin protein ligase 2; Tax1: human T-cell leukemia virus type I; TAX1BP1: Tax1-binding protein 1; TGF: transforming growth factor; TLR: Toll-like receptor; TNF: tumor necrosis factor; TNFAIP3: tumor necrosis factor α-induced protein 3; TNFR: tumor necrosis factor receptor.

## Competing interests

The authors declare they have no competing interests.

## Authors’ contributions

ELP, NVD, ALO, JHH and LAR performed the cloning of plasmids. ELP and NVD carried out real-time experiments and cultured primary cells. ELP performed the remaining experiments. ELP, JHH and RSB designed the research. JJF and CH provided technical assistance. ELP, NVD, JHH, JJL, AIL and RSB analyzed the data. ELP, JHH and RSB wrote the paper. All authors read and approved of the final version of the manuscript.
